# Advancing medical affair capabilities and insight generation through machine learning techniques

**DOI:** 10.1186/s40545-023-00670-w

**Published:** 2023-12-01

**Authors:** Karen Ka Yan Ng, Peter Chengming Zhang

**Affiliations:** University of Toronto, Toronto, Canada

## Abstract

**Background:**

Pharmaceutical companies are increasingly leveraging machine learning techniques to optimize healthcare research, drug development, and medical affairs activities. AI (artificial intelligence) tools such as chatbots, virtual digital assistants, and research tools have been explored to varying degrees of maturity in industries such as consumer goods or software technology. However, there continues to be untapped opportunities within the pharmaceutical industry to employ these technologies for enhanced engagement and education with healthcare professionals (HCPs). Pharmacists, situated at the crossroads of clinical sciences and innovation, have the potential to elevate their role and significance within the pharmaceutical industry by developing and leveraging such technologies.

**Methods:**

To address this, the python-coded tool, Medical Information (MI) Data Uses For AI Semantic Analysis (MUFASA), utilizes state-of-the-art Sentence Transformer library, clustering, and visualization techniques. MUFASA harnesses unsolicited MI data with AI technology, improving efficiency and providing actionable medical affairs intelligence for targeted content delivery to HCPs.

**Results:**

MUFASA optimizes medical affairs activities through its distinctive features: semantic search, cluster analysis, and visualization. Its proficiency in understanding inquiries, as demonstrated through 3D vector mapping and clustering tests, enhances the efficiency of MI and Medical Science Liaison (MSL) case handling. It proves invaluable in training new staff, bolstering response uniformity, and mitigating compliance risks. Leveraging the HDBSCAN algorithm, MUFASA's cluster analysis uncovers deep insights and discerns actionable themes from large inquiry data sets. The visualization graphs, generated from semantic searches, support evidence-based decisions by tracking the effectiveness of initiatives and monitoring trend shifts. Collectively, MUFASA enriches strategic decision-making, cultivates actionable insights, and bolsters healthcare professional engagement.

**Conclusion:**

There are numerous opportunities for innovation within the intersection of healthcare and data science. Pharmaceutical manufacturers, with one of their medical affairs responsibilities being the collection of unsolicited inquiries, particularly from HCPs, stand poised to leverage machine learning capabilities to optimize its processes. The abundance of data generated by the growing effort to use it in meaningful ways presents an opportunity for pharmaceutical companies to harness machine learning techniques.

## Background

The impact of machine learning techniques within the healthcare sector has become increasingly clear [1]. From insight generation to advanced search capabilities, the role of AI in the future of healthcare is promising. Pharmacist professionals employed by pharmaceutical manufacturers have a unique opportunity to leverage opportunities within the intersection between the clinical sciences and industry innovation.

This is especially compelling for medical information specialists within the pharmaceutical industry whose work relies on domain expertise, and a deep understanding of clinical therapeutic areas [2]. Medical information professionals are often responsible for the therapeutic training of industry professionals, responding to medical queries from HCPs, and generating medical affairs insights for their leadership teams. Within this area of work, aspects such as time efficiency and automation can be further improved through machine learning technology. Encouragingly, the effectiveness of machine learning techniques has already been demonstrated in other sectors such as banking, real estate, and information technology, and their application within the pharmaceutical industry presents significant opportunities [3].

With public adoption of tools such as ChatGPT, the process of searching for information is undergoing significant disruption. The ChatGPT model learns to generate texts by identifying patterns in the training data, but it lacks the capability to verify the accuracy of the information it generates. Consequently, its outputs may contain inaccuracies, errors, and false information, which is often termed as hallucination. In the context of medical affairs and medical information provided to HCPs and patients, the validation process of information generated by ChatGPT can be time consuming. As a result, other search methods like semantic search from existing documents remain popular due to their accuracy and potential for improving efficiency which have been demonstrated in various disciplines [4].

Another machine learning technique, clustering, is an example of unsupervised machine learning techniques where patterns are identified within datasets to form groups or clusters. These techniques allow users to generate insights by grouping inputs within a dataset. Within industries such as banking, this allows for customer segmentation where each individual data point can be grouped into a category based on unsupervised learning algorithms [5]. This allows for enhanced targeting by marketers. For example, data collected from a customer placing them within the “student” segment would allow automated targeting of messages from the bank isolated to student needs.

Harnessing machine learning technology within the pharmaceutical industry is an important step to meet the evolving needs of healthcare professionals. For manufacturers, the use of these new tools to improve the quality of life of their customers will provide competitive advantages. In this regard, the value proposition for developing technology that saves time and energy for HCPs is clear.

Pharmacists possess specialized expertise in medication therapy, drug information retrieval, and effective communication skills, all of which are highly relevant to the role of a medical information specialist. This paper delves into the exceptional potential demonstrated by pharmacists as they integrate elements of machine learning within the role of medical information specialists. It explores their capacity to drive innovation, highlighting the distinct and invaluable contributions they provide to the field. Furthermore, the paper examines the unsolicited value of medical information data, underscoring its significance in generating invaluable insights.

## Methods

**Fig. 1:**
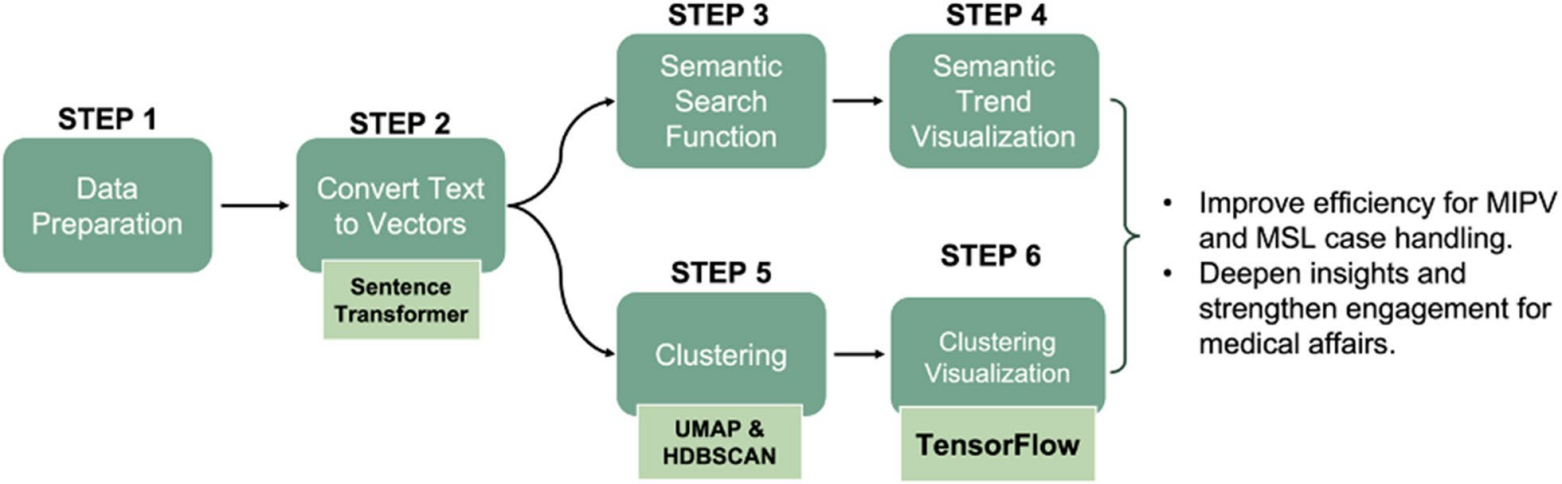
Process flow of MUFASA

The following section will explain the flow of MUFASA and provide an explanation of relevant terminology. The process flow of MUFASA that is outlined in Fig. 1 consists of four major components: data preparation (step 1), text to vectors conversion (step 2), semantic search (steps 3–4), and clustering (steps 5–6).

### Step 1: data preparation

The MI department maintains databases containing records of inquiries received over the past 10 years. The inquiries in the database cover all current LEO Pharma marketed products in the year 2022. Each entry in the database includes a case ID, date of receipt, inquiry text, inquirer’s contact information, and other relevant details. All data are exported as a single CSV file which contains 28 columns and 13,578 rows as of February 26th, 2022. The file is then processed on Jupyter Notebook, which is a platform to execute a Python program for MUFASA. A Python programming language has a widely used library called Pandas that handles CSV or any form of major table data. The CSV file is loaded by Pandas and then converted into a Pandas DataFrame. Subsequently, various data cleaning procedures are performed, such as renaming columns with simpler names, addressing missing or invalid data, and consolidating different values that denote the same entity (e.g., "DrugX" and "DrugX®" are bundled together as "DrugX").

### Step 2: convert texts into vectors

In order to facilitate semantic sentence search and cluster analysis, it is necessary to convert all sentences from the inquiry database into vectors within a high-dimensional vector space. This conversion is accomplished using a Sentence Transformer model. Among the various pretrained Sentence Transformer models available to the public, the state-of-the-art model known as all-mpnet-base-v2 is chosen due to its superior performance [6]. All inquiry texts are converted to 768-dimensional vectors by the model. During the conversion process, if an inquiry is written in French and an English translation is available, the English text will be preferred for vectorization. This ensures consistency and uniformity in the vector representation of the inquiries.

### Step 3: semantic search function tool

Following the conversion from text to vector, each vector is positioned within the embedding space based on its semantic meaning. As a result, vectors that are located in close proximity exhibit semantic similarities to the original sentences. When a user submits a search query, the Sentence Transformer model converts the query sentence into a vector and employs the k-nearest neighbors algorithm to identify other vectors situated nearby. Subsequently, the search engine returns the specified number of closest vectors to the user, along with a similarity index. These retrieved vectors represent sentences that are semantically similar to the search query and are displayed to the user as search results.

### Step 4: semantic trend visualization

MUFASA has the capability to visualize the sentences identified as semantically proximate through Semantic Search in two different formats: a list view (Fig. 3) or a chronological line plot (Fig. 4). In the list view, the N-most semantically similar sentences are displayed, accompanied by relevant metadata, including the Similarity Index and Case ID. Conversely, the line plot showcases the types of contact (HCPs, Specialists, Patients, Pharmacists) and presents the top N inquiries in chronological order, providing insights into when they were received.

### Step 5: dimensional reduction and clustering

To identify trends within the data points, a dimensional reduction from 768 to 3 is conducted using the Uniform Manifold Approximation and Projection (UMAP) algorithm. This reduction allows for the visualization of the data points in a 3-dimensional space. Among various dimensional reduction algorithms like PCA or t-SNE, UMAP is preferred due to its superior ability to handle large datasets with high dimensionality in a fast and scalable manner [7].

When a sufficient number of vector points are plotted in the 3-dimensional space, a dense group of points is referred to as a cluster. Clustering involves the task of grouping all data points into several clusters. For the MI dataset, the clustering is performed using the HDBSCAN algorithm [8]. HDBSCAN is selected over other clustering algorithms like K-means or agglomerative clustering due to its density-based nature. This means it can handle clusters with arbitrary shapes, different sizes, and densities, which is expected in datasets with reduced dimensions. As the definition of a cluster is somewhat subjective, certain parameters, such as the minimum number of data points required to form a cluster, need to be manually selected. Following the clustering process, a new column will be added to the CSV file, indicating the cluster number associated with each case inquiry.

### Step 6: visualization of clusters

**Fig. 2:**
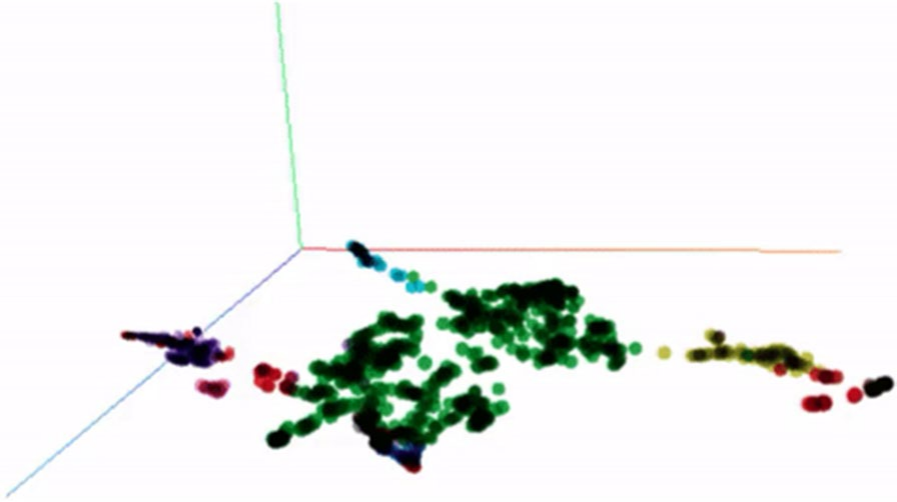
Semantic sentences are plotted in 3-dimensional space

With the data now in a 3-dimensional space, it becomes feasible to visualize the data points by assigning colors based on their respective clusters from the previous step. MUFASA utilizes TensorBoard, a visualization tool provided by Google's machine learning library TensorFlow, to display these clusters in a 3D plot [9]. In Fig. 2, the 3D visualization showcases the clusters specifically for Enstilar inquiries. Each dot on the plot represents a vector derived from an inquiry text. By hovering over a dot, users can read the corresponding inquiry and associated metadata.

## Results

### Improve efficiency for MI and MSL case handling—semantic search

**Fig. 3:**
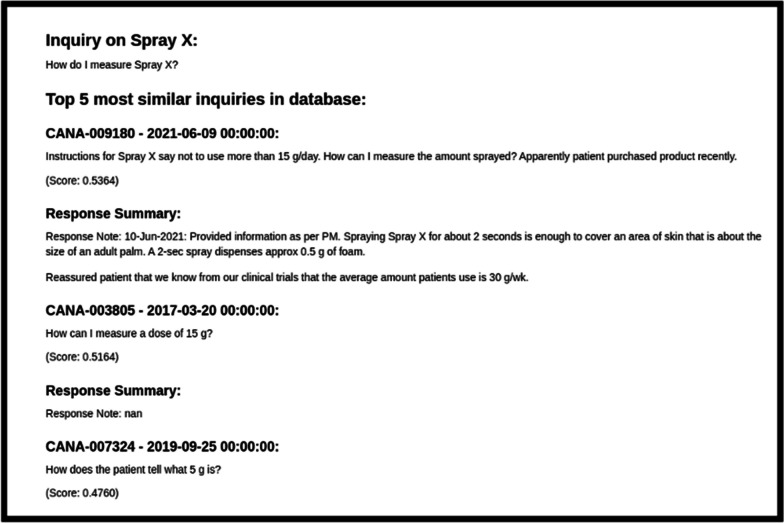
MUFASA semantic search interface—list view

Figure 3 displays a screenshot of the MUFASA semantic search interface in list view, which was executed on Jupyter Notebook. The interface presents the initial inquiry, followed by a list of semantically similar inquiries. This list includes relevant information such as case ID, date received, inquiry sentence, similarity score, and response summary. Additionally, the interface allows users to filter the results based on various criteria present in the original data, such as time, product of interest, contact type, and more. Furthermore, users have the flexibility to specify the desired number of top similar cases to be returned.

MUFASA provides a similarity index for each sentence in the returned cases, ranging from 0 to 1, where 1 indicates identical sentences. Users have the option to adjust the threshold for similarity as per their preference. It is important to note that this similarity measure is not exhaustive and may vary across different query types. Moreover, MUFASA is capable of handling multiple queries as a batch and is typo tolerant. In Table 1 below, we present a selected example of query sentences along with the top 2 similar inquiries from the past.

**Table 1:** Selected examples of semantic search queries and results of their top 2 similar sentences

Query: “How do I measure SprayX?”	
Result 1: Instruction for SprayX say not to use more than 15 g/day. How can I measure the amount with this type of formulation? Apparently patient purchased product recently	
Result 2: How can I measure a dose of 15 g?	
Query: “Stability of repackaged OintmentY”	
Result 1: How long is Ointment Y good for? Meaning shelf lifetime and time once opened?	
Result 2: What is the shelf life of Ointment Y once it has been opened?	
Query: “Big Bumps”	
Result 1: PSP reported patient experienced swelling in legs	
Result 2: PV Case report from ***-redness around his injection sites-looks like a wasp bite	

### Deepen insights and strengthen engagement for medical affairs—semantic search

**Fig. 4:**
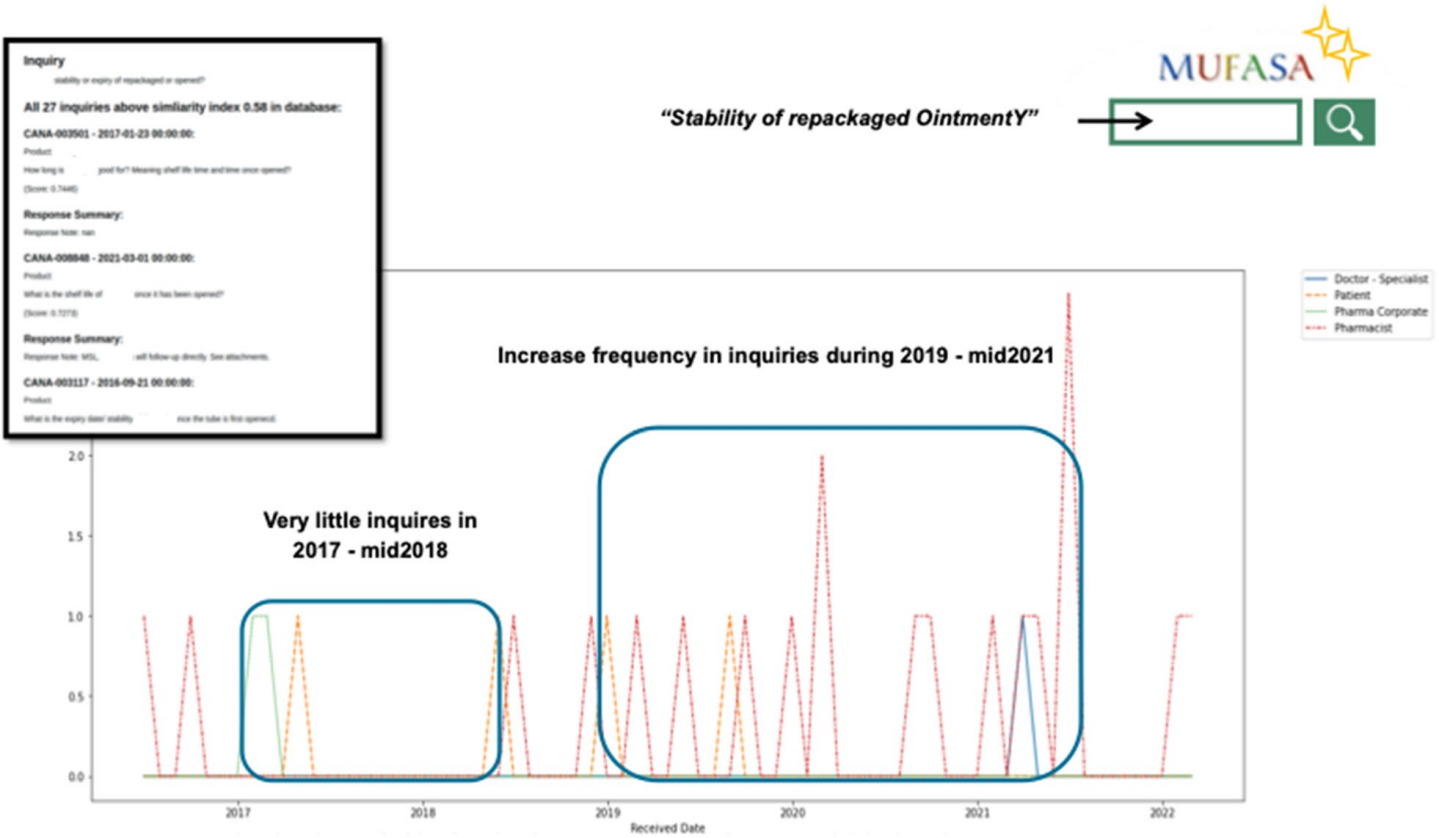
MUFASA semantic search interface—line plot view

Figure 4 showcases the line chart visualization generated seamlessly from the semantic search results, complementing the list view. In this visualization, the user manually sets the lower limit of the similarity index along with the inquiry. Consequently, MUFASA retrieves all sentences that surpass the specified lower limit of similarity index. While the list view facilitates browsing through actual inquiry texts, the line plot view enables users to visualize the frequency of received questions related to a specific topic over time. This line plot function offers various analytical capabilities, including the ability to monitor the impact of specific business decisions. In the provided example, the inquiry was set as "Stability of repackaged OintmentY".

MUFASA identified a total of 24 inquiries that exhibit semantic similarity in asking about the stability of OintmentY after it is opened. The majority of these inquiries were submitted by *patients (indicated by the orange color*) and *pharmacists (red color*). It is noteworthy that the frequency of these inquiries experienced an increase in 2019.

The surge in inquiry frequency coincided with the discontinuation of the 30 g OintmentY tube around mid-2018. Pharmacists, in response to the unavailability of the 30 g tube, seem to have resorted to repacking the 60 g product in alternative containers. However, their concerns revolve around the stability of the repackaged product.

### Deepen insights and strengthen engagement for medical affairs—cluster analysis

To better understand a large set of inquiry data, their themes or major topics need to be identified. HDBSCAN algorithm determined the following number of clusters:

· SprayX Clusters: 9

· OintmentX Clusters: 18

· OintmentY Clusters: 33

**Fig. 5:**
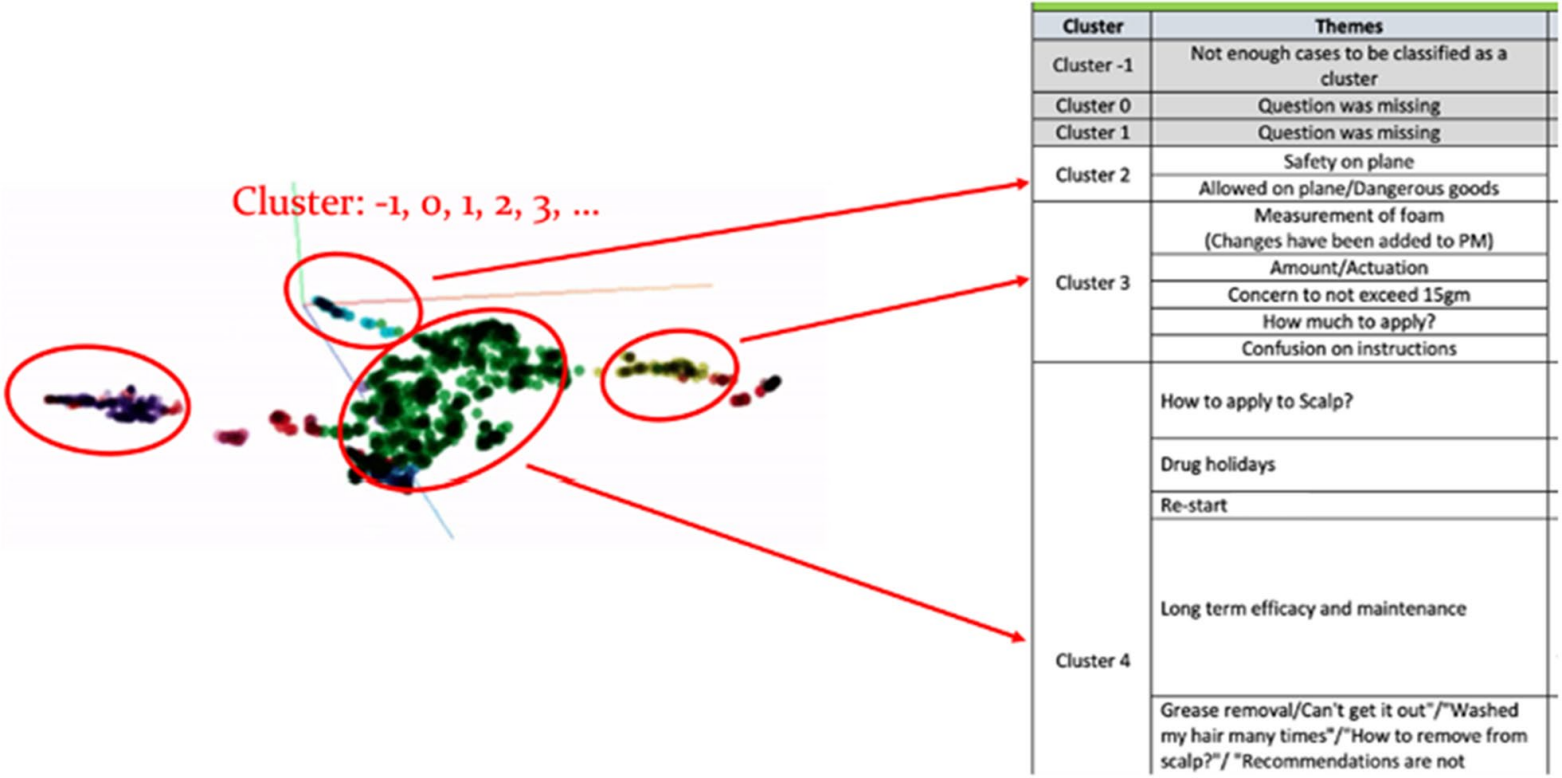
Assigning numbers to the SprayX clusters to identify themes and potential explorations

Figure 5 illustrates a diagram that explains the process of assigning numbers to the nine clusters of SprayX. In cases where datapoints do not belong to any specific cluster, they are categorized as cluster −1. Additionally, clusters with missing inquiry texts are designated as cluster 0 or 1. To simplify the identification of themes and topics, each cluster was converted to a list view instead of relying on the TensorFlow view (Fig. 1). This conversion aids in easier identification and analysis of themes within each cluster.

For the comprehensive list, the appendices include Tables 1, 2, and 3, which present the complete list of clusters for SprayX, OintmentX, and OintmentY, along with their corresponding themes. These themes are considered valuable unsolicited field insights that can provide pharmaceutical companies with potential areas for business actions.

### Validating sentence transformer’s understanding ability

**Fig. 6:**
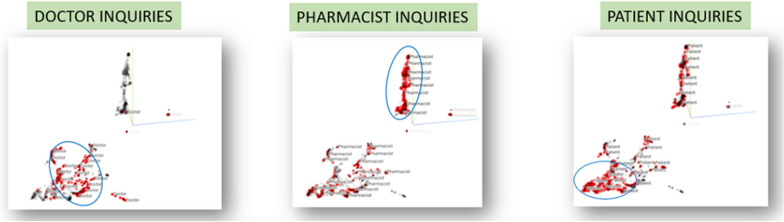
Clusters formed by inquiries from different type of contact: doctors, patients, and pharmacists

**Fig. 7:**
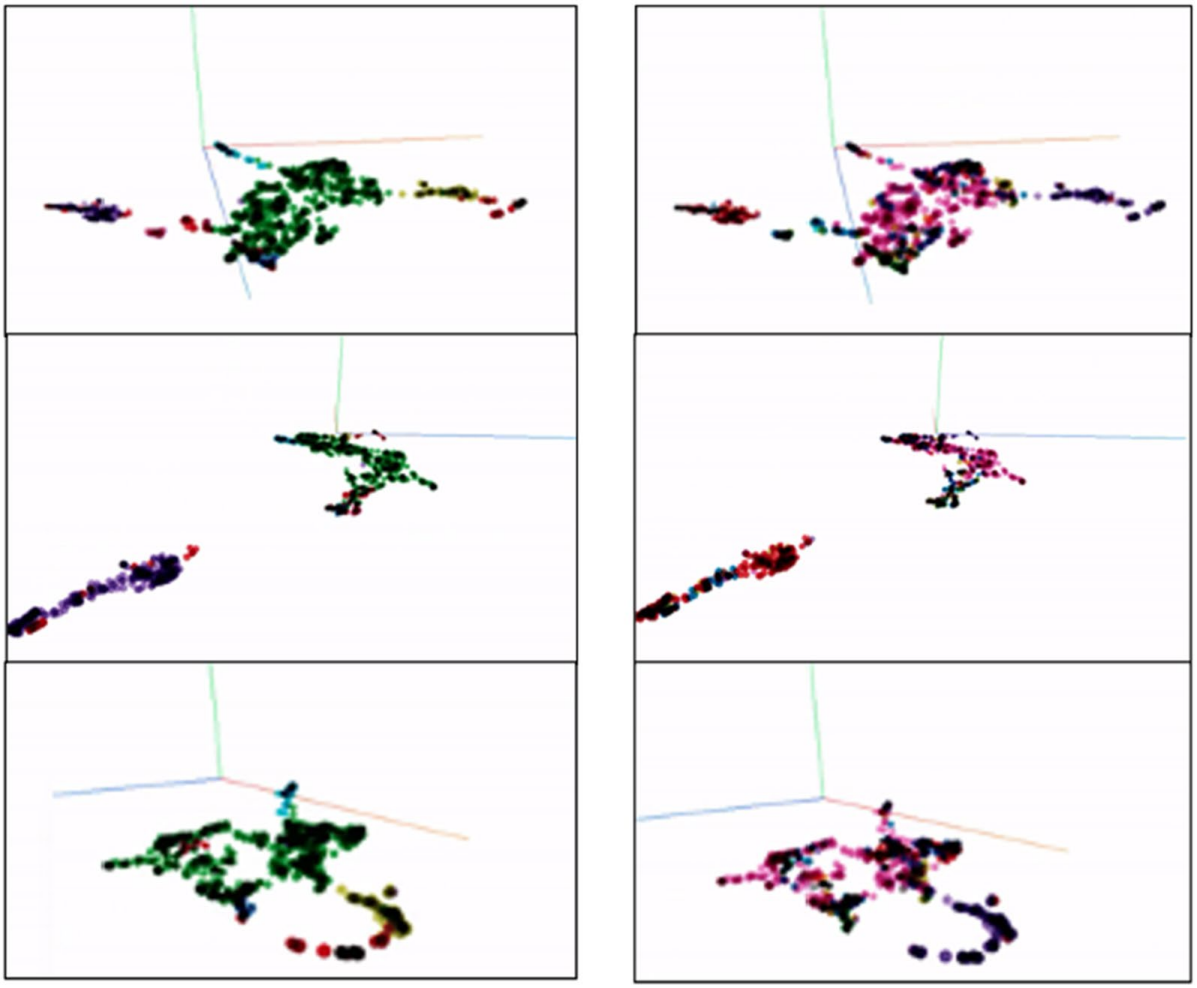
Clusters identified and colored by HDBSCAN (left) is matching the manually labeled color (right). This implies that the AI has ability to categorize/assign the clusters as accurate as manual decision

Before progressing the development of the MUFASA tool further, it is important to validate if the Sentence Transformer can semantically map the inquiries which use medical terminology. Two types of analysis were performed. Firstly, the qualitative analysis (Fig. 6), and secondly the comparison between AI clustering and clustering through the manual categorization by MI member during case handling (Request Category examples: Dose, Pregnancy, Stability, Off-label, Adverse Event) (Fig. 7).

In Fig. 6, SprayX inquiries are visualized in 3D spaces. Using the visualization tool, inquiries from doctors, pharmacists, and patients are circled in blue. The results show that the inquiries from each type of contact tend to aggregate and form clusters separately. Given each type of contact has its own distinctive interest and language use, it validates the feasibility and accuracy of the Sentence Transformer’s mapping and UMAP reduction process to organize the semantic meaning of the inquiries.

Next, by comparing the AI-determined clusters (Fig. 7-left column-AI determined cluster) with the manually determined classification (Fig. 7-right column: MI affiliate determined ‘Request Category’), one can observe the similarity in how the colors are separated. Note that because colors assigned to each cluster are randomly determined in each clustering attempt, the comparisons are made based on how the clusters are separated. The results indicate that the AI can semantically categorize the inquiry to a similar level of human interpretation.

## Discussion

### Enhancing MI and MSL case handling efficiency through semantic search

Since the development of MUFASA, the launch of advanced generative AI tools like ChatGPT and similar AI technologies have showcased the potential for the seamlessly integrated of such tools into the daily workflow. Recently, a randomized trial of generative AI marked a significant milestone where it was found that these AI tools not only accelerated task completion and led to increased productivity, but also boosted user satisfaction [10]. This underscores the evolving role of AI across multiple sectors, including healthcare.

MUFASA has been demonstrated to be an invaluable asset in the daily workflow for MI/MSL teams, where its semantic search capability enhances the accuracy of case identification, even in scenarios where the initial inquiry from physicians were vague, as demonstrated in Table 1. MUFASA's ability to provide rapid access to responses from previous similar cases has been instrumental in saving time for each MI team member. In fact, with its implementation at LEO Pharma, MUFASA has been shown to save each MI team member approximately five hours per week, assuming an average consultation time of twenty minutes per case. This, along with promoting response consistency, reducing redundancy, and mitigating compliance risks, underscores MUFASA as a compelling solution for managing routine inquiries.

### Applications of MUFASA for insight generation: the value of cluster analysis

In contrast to chatbot and generative AI solutions like ChatGPT, MUFASA is not designed for general public usage. Instead, its unique value proposition lies uniquely within the pharmaceutical industry. Though both tools stem from the broader field of AI, they each provide distinctly different capabilities. ChatGPT, for example, excels at generating responses based on existing data, while MUFASA offers a unique feature enabling users to visually map the relationships of inquiries and discern thematic clusters—a functionality not offered by ChatGPT or similar tools. This key difference allows MUFASA to encourage user-driven interpretation of themes and detection of business trends, which might necessitate intuitive skills not yet fully encapsulated when using tools such as ChatGPT. Thus, MUFASA accentuates the crucial role of medical affairs personnel instead of replacing it.

MUFASA's analytical system, which clusters inquiries, offers a profound understanding of the interests and questions of HCPs. In conjunction with insights from practicing pharmacists and PharmD residents, MUFASA paves the way for strategies that can be readily implemented, as exemplified in Tables 1, 2, and 3. Utilizing the comprehensive knowledge and patient counseling skills of these professionals, the trends identified by MUFASA can be translated into practical solutions, such as the development of pharmacist-oriented educational materials and bespoke content for MI websites.

MUFASA also serves as an agent for continuous improvement within pharmaceutical companies. Its ability to track trends allows for immediate adjustments to packaging or educational materials in response to changing needs. Moreover, its geographic visualization feature enables professionals to adapt their strategies to align with regional healthcare practices and patient requirements.

Furthermore, MUFASA's cluster analysis function is advantageous to the broader Medical Affairs department. The MI department can leverage unsolicited inquiries to obtain a broader perspective, eliminating potential sampling and selection bias that can be observed from advisory boards. The insights acquired from the MI database can aid in addressing concerns raised by healthcare professionals regarding new drugs, additional data needs, or recent studies. This tool thus can also contribute to the planning of Other Learning Activities or substantiate the need for such programs.

Ultimately, MUFASA's cluster analysis capability offers an efficient way of extracting valuable insights from unsolicited medical information. In the past, text data quantification and theme identification was a challenging task. Keyword and category searches often fell short due to language variations and potential inconsistencies in categorizing inquiries by different personnel. With MUFASA, the process of identifying trends becomes significantly less labor-intensive. This shift enables the MI team to concentrate more on specialized inquiries, thereby promoting a more comprehensive understanding of data and enabling the generation of actionable insights. As a result, this supports proactive departmental training initiatives, leading to increased value delivery by pharmaceutical companies.

### Limitation and future of MUFASA

As a machine learning tool, MUFASA’s ability is fundamentally limited by the volume and quality of data available. In addition, semantic search results can be affected by variations in wording and phrasing, and those effects on the results are not clear at this moment. Similarly, what is considered clinically relevant may not be able to be processed by the Sentence Transformer in identifying relevant “similar cases”. Medical terminology and dictions are complex and nuanced, but Sentence Transformer models are trained based on usual language setting and not trained for medical situations. Therefore, while there may be an interest in training them from MI case handling to better understand how cases are categorized, there is an argument to not train the machine learning model for specific identification of clusters. This is because there is benefit in the lack of training as a lack of training removes the bias for which an individual thinks what a cluster should be.

It is also important to mention that semantic search does not factor in sentiment analysis. Continual development of MUFASA should explore the integration of AI’s ability for sentiment analysis as it is also important for a pharmaceutical company to analyze their social share of voice by sentiment and topic at the same time. For example, although a product may have a high social share of voice (SOV), it is important to address any negative comments.

For the cluster analysis, it was observed that one cluster may contain multiple themes. However, the purpose of clustering is to have the AI help identify themes that are not difficult to tease out manually. Although clusters are not fully isolated through the clustering process, separating the information from thousands of inquiries to smaller organized clusters of hundred inquiries allows for an easier identification of topics. Therefore, the utility of MUFASA ultimately made the process more efficient and effective despite such limitations.

MUFASA is still in its infancy in development and lacks integration with other data ecosystems at LEO Pharma. For example, the addition of geographical data can add tremendous potential to MUFASA. Geographical analysis will provide LEO Pharma a way to measure its breadth of reach of scientific messages in comparison to its competitors. When geographical locations are added to other demographic filters, such as gender, age, or occupation to identify important target audiences, this could lead to unique advantages against LEO Pharma’s competitors. The future of MUFASA should also explore the addition of other ecosystems that will allow the tool to evaluate large volumes of publications, clinical trials, and text insights from advisory boards which can help quickly identify, discern any new key topics of interest for HCPs.

## Conclusion

In conclusion, the potential to increase productivity through machine learning tools within the pharmaceutical industry is becoming increasingly demonstrated. For medical affairs professionals such as pharmacists within the industry, there may be opportunities to integrate machine learning technologies with clinical tasks, and such professionals may consider upskilling in areas such as data science. MUFASA as a tool has illustrated the potential to optimize current processes within the medical information function. Through investment and commitment from pharmaceutical manufacturers, the continued advancement of tools such as MUFASA will bring new opportunities and drive efficiency in solving new and existing challenges.

## Data Availability

The data that support the findings of this study are available from LEO Pharma, but restrictions apply to the availability of these data, which were used under license for the current study, and so are not publicly available. Data are however available from the authors upon reasonable request and with the permission of LEO Pharma.

## Appendix

SprayX cluster themes (see Tables 2, 3, and 4).

**Table 2:** SprayX cluster themes identified by HDBSCAN algorithm

SprayX cluster themes		
Cluster	Themes	Potential explorations
Cluster -1	Not enough cases to be classified as a cluster	N/A
Cluster 0	Question was missing	N/A
Cluster 1	Question was missing	N/A
Cluster 2	Safety on plane	Med info-website potential, educate pharmacists
	Allowed on plane/dangerous goods	
Cluster 3	Measurement of foam (Changes have been added to PM)	# Inquiry Vs Time to see if # of questions decreased (Are the PM changes sufficient)
	Amount/actuation	
	Concern to not exceed 15gm	
	How much to apply?	
	Confusion on instructions	
Cluster 4	How to apply to Scalp?	Add content to Med Info Website (therein forth described as “Med Info Website”)
	Drug holidays	Med Info Website
	Re-starting SprayX	Med Info Website
	Long term efficacy and maintenance	Med Info Website (SprayX-Data) #Inquiry vs time to see if # of questions changes following a) rep reactive handling of HCP requests for long-term/maintenance use; b) PM changes
	Grease removal/Can't get it out"/"Washed my hair many times"/"How to remove from scalp?"/ "Recommendations are not working"	Sending a follow-up questionnaire if the "Shampoo on dry hair” or general recommendations works after reply
	Information in the use of SprayX post-4 weeks	Med Info Website (SprayX Data)
	Disposal of SprayX	Educate Pharmacists, Doctors, Med Info Website
	Paul Study: SprayX vs DrugX/ SprayX and DrugY^®^	Med Info Website (SprayX- Data)
	SprayX Study: SprayX vs DrugZ/ SprayX and DrugZ	Med info Website (SprayX-Data)
	How long to leave application on? / How long does it take to absorb?	Educate Pharmacists (part of counselling)
	Use with vitamin D	Med Info Website
	Use in adolescence/use in children	Med Info Website
	Confusion on instructions of "A can lasts 4 days" with their physician’s SIG	PM improvements in wording
	Application on off-label sensitive areas ("Can it be applied on […]” (Areas where PM advises not to use)	Med Info Website
	SprayX vs DrugA/SprayX and DrugA	Med Info Website
	Use in pregnancy/breastfeeding	Med Info Website
	Putting on gloves/socks/occluding area (band-aid) after application from patients/"used under occlusion"	Educate pharmacists (part of counselling)
	Calcium levels	Med Info Website
	SprayX vs phototherapy/ SprayX and phototherapy	Med Info Website
	Staining fabrics	Educate pharmacists (part of counselling)
	Removal of product. ("Do I wait for it to dry?", "Do I need to wipe off the excess?")	Educate pharmacists (part of counseling)
	SprayX trial data	Med Info Website (SprayX Data)
	Any concerns with the treatment of hair (colored hair/perm)	Educate pharmacists (part of counselling), Med-Info Website
	Risks of inhalation	Educate pharmacists (part of counseling)
	Use of moisturizer/sunscreen	Educate pharmacists (part of counseling)
	Psoriasis of various kinds (Pustular psoriasis, plaque psoriasis, palmoplantar psoriasis, nail psoriasis)	Med Info Website
	Does it contain "…"	Med Info Website
	Positive feedbacks/marketing feedback	Data visualization: use of MAFUSA
	Hyperpigmentation	Med Info Website
	Photocarcinogenicity/sun exposure	Educate pharmacists (part of counselling)
	Is SprayX the same as DrugB^®^	Med Info Website
	Accidentally got into eyes	Educate pharmacists (part of counselling)
	Requests for OLA slide decks	Data visualization: use of MAFUSA
Cluster 5	Appearance/"why is the foam not foaming?"	Product monograph improvements in wording
	Cold feeling after application	Med Info Website
	Product defects	Data visualization (Use of MAFUSA)
	Application near the eyes	Med Info Website
	What is the environmental impact of improper disposal?	Educate pharmacists, doctors, Med Info Website
	Compassionate use	Med Info Website (instructions/forms)
Cluster 6	SprayX patient cases program: use beyond 4 weeks	N/A: Very old cases
Cluster 7	PV case reports—off-label use	Data visualization: use of MAFUSA

OintmentX cluster themes.

**Table 3:** OintmentX cluster themes identified by HDBSCAN algorithm

OintmentX cluster themes		
Cluster	Themes	Potential explorations
Cluster -1	Not enough cases to be identified as a cluster ex: stability once opened (Pharmacists)	NA
Cluster 0	Question was missing	NA
Cluster 1	Question was missing	NA
Cluster 2	Sample requests	Data visualization: MUFASA (Geographical explorations in future)
Cluster 3	MSDS	Data visualization: MUFASA
Cluster 4	MSDS (Mainly from pharmacists)	Data visualization: MUFASA
Cluster 5	Does it contain "…" (Ingredients)	Med Info Website
Cluster 6	PV cases (AE, Off Label)	Data visualization: MUFASA
Cluster 7	Product defects (cracks to the tube)	Data visualization: MUFASA
Cluster 8	Safety inside mouth	Med Info Website
	Can it be applied to inside of the noses	Med Info Website
	Use of it more than 14 days	Med Info Website
Cluster 9	MRSA coverage w/OintmentX	Med Info Website
	Requests for OLA slides	Data visualization: MUFASA
Cluster 10	Availability (Backorder)	N/A
Cluster 11	Availability of pack size of 15 g	Compare it with in-use stability
Cluster 12	MSDS requests	Med-Info Website
Cluster 13	Receiving documents	N/A
Cluster 14	Receiving samples	Geographic visualization
Cluster 15	Why is there a 3-month expiry date? (patients/pharmacists)	Med Info Website
Cluster 16	In-use stability after repackaging	Compare it with inquiry for availability of 15 g inquiry

OintmentY^®^ cluster themes.

**Table 4:** OintmentY cluster themes identified by HDBSCAN algorithm

OintmentY cluster themes		
Cluster	Themes	Potential explorations
Cluster -1	Not enough cases to be identified as a cluster: Price is too expensive	N/A
Cluster 0	Question missing	N/A
Cluster 1	Question missing	N/A
Cluster 2	Market research- Off label use not caused by atopic dermatitis	N/A
Cluster 3	Question missing	N/A
Cluster 4	Requests for slide decks	Data visualization: MUFASA
Cluster 5	Does OintmentY contain "…" (Ingredients)	Med Info Website
Cluster 6	Request for dosing information in children < 2 years old	Med Info Website
Cluster 7	Facial flush use with alcohol	Med Info Website, Educate pharmacists (part of counselling)
Cluster 8	Availability of OintmentY	Date visualization: MUFASA (Compare it with inquiry of stability once opened questions)
Cluster 9	Off label use with vitiligo	Data visualization: MUFASA
Cluster 10	Is there a Compassionate Use Program (CUP)?	Med Info Website (Instructions, Forms)
Cluster 11	CUP process	
Cluster 12	Off label use in sensitive area (eyes, face, gluteal cleft)	Data visualization: MUFASA
Cluster 13	Maximum dose/maximum duration to use	Med Info Website
Cluster 14	PV-AE types of report	Data visualization: MUFASA
Cluster 15	PV-AE Types of report with PSP# provided	Data visualization: MUFASA
Cluster 16	Patient experiencing AE and wanting reimbursement	Data visualization: MUFASA
Cluster 17	OintmentY use off label- inside mouths	Data visualization: MUFASA
Cluster 18	Can it be used in sensitive reproductive areas? (genitals)	Med Info Website
Cluster 19	Requests for OintmentY/DrugC Study	Med Info Website #Inquiry vs time to see if # of questions change following a) rep handling of long-term safety/cancer warning message with Trial data b) removal of boxed warning
Cluster 20	Request for Off label use data for Vitiligo	Data visualization: MUFASA
Cluster 21	Request for information for off-label uses in other types of areas	Data visualization: MUFASA
Cluster 22	The use of OintmentY with/without steroids + Why is OintmentY second line?	Med Info Website
Cluster 23	Phototherapy/sunlight	Med Info Website, educate pharmacists (part of counselling)
Cluster 24	Use on eyelids/getting into the eyes	Med Info Website, educate pharmacists (part of counselling)
Cluster 25	Malignancy/cancer risks	Med Info Website Changes in inquiry frequency over time to see if PM change (removal of boxed warning) has had an impact
Cluster 26	Safety concerns: renal failure	Med Info Website
Cluster 27	Product defect: difficulty getting ointment out of tube	Product feedback
Cluster 28	Request on information for the use on children under the age of 2	Med Info Website
Cluster 29	Request on information for the use on children under the age of 2 (provides strength in questions)	Med Info Website
Cluster 30	In-use stability of tube once opened	Data visualization: MUFASA (Has it been increasing?)
Cluster 31	Doctor recommends putting tube in fridge	Opportunities to educate doctors/pharmacists to know that the stability is compromised once put in fridge Med Info Website should upload information about refrigeration and stability

## Abbreviations

ChatGPT: Chat generative pre-trained transformer
CSV: Comma-separated values
HCPs: Healthcare professionals
HDBSCAN: Hierarchical density-based spatial clustering of applications with noise
ID: Identity
MI: Medical information
MSL: Medical science liaison
MUFASA: Medical information data uses for AI semantic analysis
PCA: Principal component analysis
t-SNE: T-distributed stochastic neighbor embedding
UMAP: Uniform manifold approximation and projection
